# Effects of Ca Substitution in Single-Phase Sr_1-x_Ca_x_Ti_0.8_Fe_0.2_O_3-ẟ_ Oxygen Transport Membranes and in Dual-Phase Sr_1-x_Ca_x_Ti_0.8_Fe_0.2_O_3-ẟ_-Ce_0.8_Gd_0.2_O_2_ Membranes

**DOI:** 10.3390/membranes15090258

**Published:** 2025-08-29

**Authors:** Veronica Nigroni, Yuning Tang, Stefan Baumann, Doris Sebold, Enrico Malgrati, Paolo Fedeli

**Affiliations:** 1 Department of Chemistry, University of Parma, 43121 Parma, Italy; veronica.nigroni@unipr.it; 2 Institute of Energy Materials and Devices IMD-2, Forschungszentrum Jülich, 52425 Jülich, Germany; y.tang@fz-juelich.de (Y.T.); s.baumann@fz-juelich.de (S.B.); d.sebold@fz-juelich.de (D.S.); 3 Materials and Generation Technologies Department, Ricerca sul Sistema Energetico-RSE SpA, 20134 Milan, Italy; enrico.malgrati@rse-web.it

**Keywords:** oxygen transport membranes, dual phase, strontium titanate

## Abstract

Oxygen transport membranes (OTMs) have gained a lot of attention for their application in different innovative fields, but the development of new materials able to combine high oxygen permeability and good chemical stability is crucial to boost the exploitation of such membrane-based technologies. Perovskite oxides are widely studied as mixed ionic-electronic conductors for the realization of OTMs. In this article, we focus on _Sr1-x_Ca_x_Ti_0.8_Fe_0.2_O_3-ẟ_ (SCTF) perovskites and investigate the effect of Ca content on the A-site of the permeation properties, both in single-phase SCTF membranes and in dual-phase membranes obtained by combining SCTF and the ionic conductor Ce_0.8_Gd_0.2_O_2_ (CGO). In single-phase samples, we observed that the substitution of 40% Ca preserves the permeation performances of the non-substituted SrTi_0.8_Fe_0.2_O_3−ẟ_ membrane while allowing for a substantial decrease in the sintering temperature, thus facilitating membrane manufacturing. In dual-phase membranes, the increase in the Ca content in the perovskite causes an increase in grain size. The permeation is, at least partially, controlled by the kinetics of the surface exchange reactions. This limitation can be overcome by the addition of an activation layer; however, the permeance of activated CGO-SCTF membranes still remains lower compared to the single-phase parent perovskitic membranes.

## 1. Introduction

Oxygen transport membranes (OTMs) are ceramic structures that allow for the selective permeation of oxygen through the solid-state diffusion of oxygen ions, provided that the temperature is sufficiently high (typically T > 750 °C) and an oxygen partial pressure gradient across the membrane is maintained [1,2,3].

Due to their properties, OTMs have been proposed for several applications, including pure oxygen generators for oxy-fired industrial combustion [4,5,6,7] and catalytic membrane reactors (CMRs) to produce chemical commodities and energy vectors [8,9].

**Figure 1:**
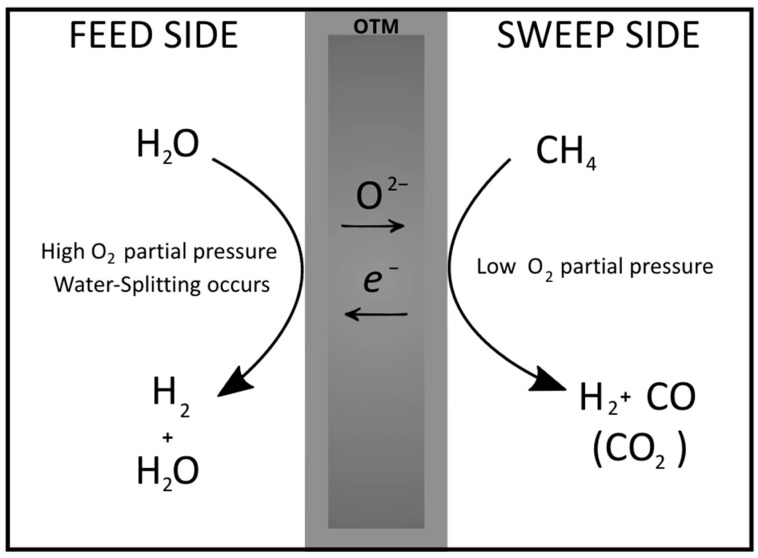
Schematization of the operation of a CMR for thermal water splitting coupled with the partial oxidation of methane. In the membrane, the arrows indicate the transport of oxygen ions and electrons.

Membrane reactors realized with OTMs can be used to produce pure hydrogen from water, a clean, carbon-free, and renewable resource, through thermochemical water splitting (TWS) [10,11,12,13]. Due to the in situ oxygen removal, CMRs allow a shift in the thermodynamic limitations of the water-splitting process towards the products [14,15,16,17]. The image in Figure 1 represents an example of how such devices work. On one side, vapour is fed, and the oxygen produced by H_2_O splitting is continuously removed by the membrane. On the other side of the membrane, a reducing gas can be supplied to keep a high O_2_ partial pressure gradient [18,19,20]. To achieve a high process intensification, TWS at the feed side can be coupled with the partial oxidation of methane (POM) at the permeate side, a reaction that produces syngas (H_2_ + CO). The controlled and uniform oxygen supply through the membrane prevents the formation of hot spots and the total oxidation of CH_4_ to CO_2_ [21].

For application in CMRs, OTMs must exhibit both high oxygen fluxes and sufficient chemical stability at high temperatures in reducing atmospheres. Therefore, a focus on the materials used for the membranes is required. Perovskite crystals belonging to the class of mixed ionic electronic conductors (MIECs) are one of the most studied materials for OTMs [22,23,24,25,26], since they have high ionic conductivity and can conduct electrons simultaneously. MIECs can be used to prepare single-phase membranes or can be coupled to another compound to obtain composite dual-phase membranes made of a predominant ionic and a predominant electronic conductor. Dual-phase membranes usually show higher stability but lower permeability than single-phase ones [27,28]. The leading MIEC materials for OTMs are La_x_Sr_1-x_Co_y_Fe_1-y_O_3−δ_ and Ba_x_Sr_1-x_Co_y_Fe_1-y_O_3−δ_ perovskites, as they show high permeability [29,30,31,32]. However, they have low stability under reaction conditions [33,34,35]. On the basis of such composites, many investigations have been carried out with the aim of finding more stable materials without affecting their permeability. Recently, SrTi_1-x_Fe_x_O3 (STF) has been proposed as a promising material class, as it shows both good resistance in reducing atmospheres and high oxygen fluxes [36,37,38]. However, tests in different conditions have highlighted the tendency to form carbonates when exposed to CO_2_ due to the presence of strontium on the A-site of the perovskite [21,39]. Xi et al. showed that in Sr**_x_**Fe**_1.5_**Mo**_0.5_**O**_6_**_−**δ**_ perovskite oxides, high Sr content led to a facilitated formation of SrCO**_3_** on the surface [40]. Further studies revealed that the A-site cation substitution of Sr with a smaller element like Ca can improve CO_2_ tolerance, but the effect of Ca concentration on the permeation properties was not investigated [23].

In this study, we focus on the characterization of the oxygen separation performances in Sr_1-x_Ca_x_Ti_0.8_Fe_0.2_O_3−δ_ with different Ca contents. In particular, we compared the parent SrTi_0.8_Fe_0.2_O_3−δ_ material without substitution with the Ca-substituted A-site with 10% (Sr_0.9_Ca_0.1_Ti_0.8_Fe_0.2_O_3−δ_) and 40% (Sr_0.6_Ca_0.4_Ti_0.8_Fe_0.2_O_3−δ_).

Sr_1-x_Ca_x_Ti_0.8_Fe_0.2_O_3−δ_ compounds with different Ca substitutions were also studied in combination with an ionic conductor to form dual-phase membranes. Therefore, SrTi_0.8_Fe_0.2_O_3−δ_, Sr_0.9_Ca_0.1_Ti_0.8_Fe_0.2_O_3−δ_ and Sr_0.6_Ca_0.4_Ti_0.8_Fe_0.2_O_3−δ_ were combined with the ion-conducting fluorite Ce_0.8_Gd_0.2_O_2_ (CGO20). Membranes were prepared with a weight ratio of 40% perovskite and 60% fluorite.

## 2. Materials and Methods

### 2.1. Sample Preparation

The perovskites SrTi_0.8_Fe_0.2_O_3−δ_ (STF82), Sr_0.9_Ca_0.1_Ti_0.8_Fe_0.2_O_3−δ_ (SCTF9182), and Sr_0.6_Ca_0.4_Ti_0.8_Fe_0.2_O_3−δ_ (SCTF6482) were prepared through a solid-state reaction mechanism. The raw materials SrCO_3_ (Sigma-Aldrich, St. Louis, MO, USA), Fe_2_O_3_ (Sigma-Aldrich), TiO_2_ (Riedel-de Haën, Seelze, Germany), and CaCO_3_ (Sigma-Aldrich) were added in stoichiometric amounts into a PE bottle with milling balls (yttrium-stabilized zirconia, YSZ, 5 mm diameter) and ethanol. The mixture was prepared with a powder/ethanol/ball ratio of 1:2:3. The bottle was then put on a rotating bench for 48 h at 60 rpm. After mixing, the milling balls were sifted and ethanol was evaporated in a drying chamber at 70 °C. The powder was calcined at 1075 °C in an alumina crucible for 25 h. The powder was then again milled on a rolling bench for 48 h with YSZ milling balls and ethanol, achieving the desired particle size distribution. Dual-phase powders were prepared by mixing respective amounts of Ce_0.8_Gd_0.2_O_1.9_ (CGO) (Treibacher Industries AG, Althofen, Austria, 99%) with the self-synthesized powders STF82, SCTF6482, and SCTF9182. The powders were also mixed with YSZ balls in ethanol, as described above.

Membrane samples were prepared by pressing the powders in a 20 mm diameter stamp at 15 kN pressure for 1.5 min. After dilatometry analysis, the sintering of the membranes was carried out, as outlined in the table below. The sintering temperatures were decided by studying the sintering behaviour of each material in order to obtain dense membranes.

### 2.2. Characterization

The crystal structure of the materials prepared were studied by X-ray diffraction (XRD) carried out with a D4 ENDEAVOR (Bruker, Karlsruhe, Germany) diffractometer. The diffraction angle varied between 10° and 85° 2θ. The diffractograms were then qualitatively analyzed by the programme X Pert High Score (PANalytical, Almelo, The Netherlands).

The morphologies of the dual-phase membranes were studied by field emission-scanning electron microscopy (FE-SEM) using a Zeiss Gemini SEM 450 (Carl ZeissNTS GmbH, Oberkochen, Germany) microscope. The porosity of the investigated samples was calculated by image analysis on cross-sectional micrographs using the software Nikon NIS-Elements 5.21.02.

The oxygen permeation measurements were carried out on membranes prepared by pressing the respective powders into discs, which were sintered and subsequently ground and polished to a thickness of ≈1 mm and a diameter of ≈14.7 mm. The experimental device consists of a vertical quartz glass housing in which the discs are sealed on both sides with a gold ring. The free membrane surface has a diameter of 13 mm. The feed side was fed by ambient air at 250 ml_N_ min^−1^ flux. Argon was used as the sweep gas at a flow rate of 50 ml_N_ min^−1^. The permeated oxygen between 1025 °C and 700 °C in 50 K-steps was detected with a mass spectrometer (Omnistar, Pfeiffer Vacuum, Hessen, Germany). By simultaneous quantification of permeated nitrogen, it is possible to deduce leakage through the membrane or sealing. The oxygen permeation through the membrane considering leakage can be calculated according to the following equation:

(1) $$j_{O2}=F_{Ar}\left(\frac{X_{O2}-\frac{1}{4}X_{N2}}{1-X_{O2}-X_{N2}}\right)\frac{1}{A_{mem}}$$

where ***F_Ar_*** is the argon flow rate, ***X*_O2_** and ***X*_N2_** are the concentrations of oxygen and argon in the permeate gas, ***A_mem_*** is the area of the membrane, and the factor $\frac{1}{4}$ represents the oxygen/nitrogen ratio assuming that the leak is non-selective. Since the driving force for the permeation of oxygen, the oxygen partial pressure, is temperature-dependent and the thickness of the sample has an experimental error to the desired 1 mm of thickness, the material’s permeance must be normalized to the reference. The permeance of the membrane is calculated assuming Wagner’s behaviour by the equation below:

(2) $$Permeance=\frac{j_{O2}}{ln\frac{{p’}_{O2}}{{p’’}_{O2}}}\frac{L_{mem}}{L_{0}}$$

where ***p’_O_*_2_** and ***p’’_O_*_2_** are the oxygen partial pressures in the feed and sweep sides, ***L_mem_*** is the actual thickness of the membrane; and ***L_0_*** is the reference thickness of 1 mm.

## 3. Results and Discussion

### 3.1. Single-Phase Materials

#### 3.1.1. Microstructure

**Figure 2:**
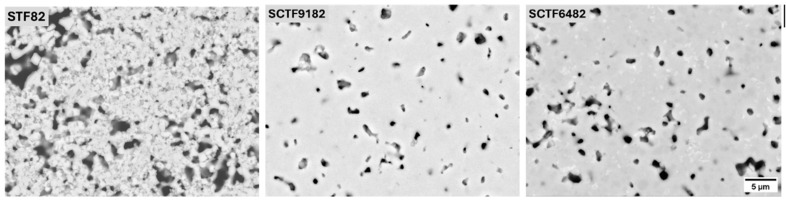
SEM micrographs of polished cross-sections of the sintered membranes composed by STF82, SCTF9182, and SCTF6482.

The microstructure of the sintered membranes was investigated by means of SEM. Figure 2 displays the cross-sectional images of the samples STF82, SCTF9182, and SCTF6482. A certain amount of residual porosity is detectable in all samples, being higher in the non-substituted STF82 than in Ca-containing SCTF9182 and SCTF6482. The estimated porosity fraction was 23%, 5%, and 9% for STF82, SCTF9182, and SCTF6482, respectively. However, all the membranes were sufficiently dense to provide gas tightness (cf. Section 3.1.3). Although the sintering temperature was reduced with increasing Ca substitution without extending the dwell time (see Table 1), a comparable microstructure was achieved in all membranes. This confirms that the introduction of Ca on the A-site of Sr_1-x_Ca_x_Ti_0.8_Fe_0.2_O_3−δ_ has a beneficial effect on densification during sintering, as already reported for similar materials [41,42].

#### 3.1.2. Crystal Structure

**Figure 3:**
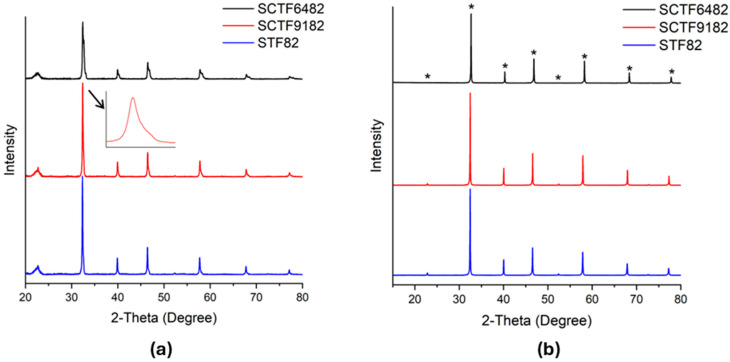
Diffractograms of (**a**) prepared STF powders after calcination at 1075 °C for 25 h and (**b**) STF membranes after sintering at 1450 °C for STF82, 1400 °C for SCTF9182, and 1350 °C for SCTF6482 for 5 h. Inset in Figure 3a shows an example of peak twinning in SCTF9182 powder sample. The asterisk highlights the wanted peaks for these materials.

XRD analysis was used to monitor the crystal structure of the powders (Figure 3a) and the sintered membranes (Figure 3b). The crystal structure of the calcined perovskites is mainly cubic; however, it is possible to note a peak twinning in the calcined materials with Ca partially substituted, revealing the presence of an additional perovskite phase with tetragonal symmetry. This indicates the possibility of an incomplete or intermediate phase formation during the calcination process.

In contrast, the diffractograms after sintering show the presence of a single cubic perovskite phase without peak twinning for all compositions, confirming that the final heat treatment completes the intended phase formation. For perovskite-based OTMs, the cubic symmetry is the most desirable, since it is associated with a higher mobility of oxygen vacancies compared to more distorted crystal lattices due to a higher degree of isotropy in oxygen site spatial distribution and better geometrical capability for ion hopping [4,43,44,45,46]. The lattice parameters of sintered membranes were estimated by Rietveld refinement of the XRD patterns in Figure 3b (see Figure S1) and are 3.9041(0) **Å**, 3.8998(3) **Å,** and 3.8800(1) **Å** for STF82, SCTF9182, and SCTF6482, respectively. The decrease in lattice parameter upon increasing calcium fraction is consistent with the lower Shannon radius of Ca^2**+**^ with respect to Sr^2**+**^ [47].

#### 3.1.3. Oxygen Permeation Performance

The gas tightness of the sintered membranes was checked at room temperature by means of helium leakage tests. All the samples showed a leakage < 10^−6^ mbar·L·s^−1^·cm^−2^, corresponding to sufficient gas tightness.

**Figure 4:**
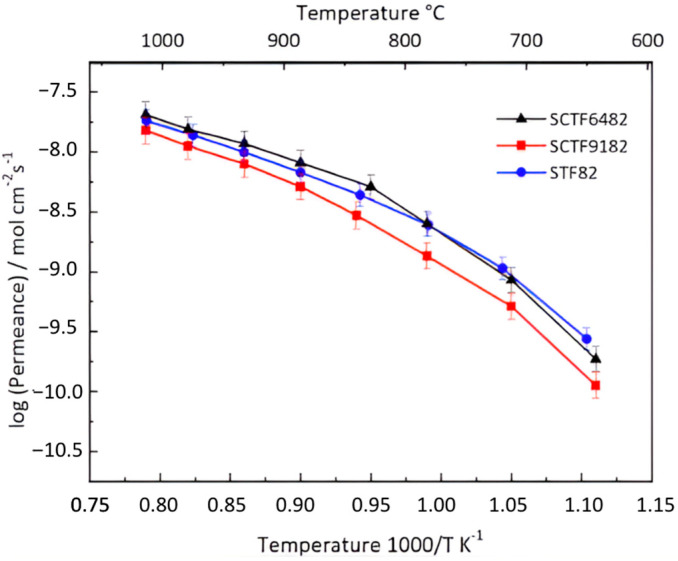
Oxygen permeance of STF82, SCTF9182, and SCTF6482 at decreasing temperatures from 1000 °C to 750 °C.

The permeance of the single-phase membranes, normalized to the membrane thickness according to Equation (2), is shown in Figure 4. The experimental error is approximately 10%. The oxygen permeation tests were repeated twice for reproducibility, and the data were confirmed. STF82 shows a permeance comparable to the one previously observed on membranes with similar compositions [36]. The comparison among the three different membranes suggests that Ca substitution affects the oxygen permeation efficiency only by a small extent. Indeed, a small substitution of Ca (i.e., 10 mol% in SCTF9182 sample) causes a slight decrease in the performance of the membrane, while when the substitution is greater (i.e., 40 mol% in SCTF6482 sample), the performance is comparable to the non-substituted membrane. Surprisingly, the permeation curves shown in Figure 4 suggest that lower amounts of Ca substitution have a higher effect on the permeance than higher Ca fractions. Therefore, a clear effect of the Ca content on the oxygen transport capability cannot be inferred from these measurements, apart from the consideration that even in the case of the lowest performing SCTF9182 membrane, the introduction of calcium does not lead to a dramatic reduction in the permeation compared to the unsubstituted STF82 sample. To obtain deeper insight into the role of Ca in the permeation process, the difference among the performance of the three investigated compositions should be emphasized. A possible strategy, which will be the focus of future work, is the preparation and characterization of thin (<30 µm) supported STF82, SCTF9182, and SCTF4682 membranes, where the optimized microstructure should involve higher permeation fluxes and, possibly, a more obvious effect of calcium. Additionally, computational studies could be of great help to better understand the influence of the Ca fraction in the perovskite lattice on the ionic transport.

From the Arrhenius plots in Figure 4, it is possible to extract the activation energies, which are reported in Table 2.

The obtained values are similar for the three compounds, indicating that the permeation is mainly controlled by the same mechanism in all samples. For thick (~1 mm) dense membranes, such a limiting step is typically assigned to the ionic transport in the bulk. The activation energy is slightly lowered at higher Ca substitutions, which is coherent with the observed trend of the permeance.

Previous studies [41] showed that the introduction of Ca in the A-site of the SrTi_0.8_Fe_0.2_O_3−δ_ perovskite can confer some beneficial effects, such as the improvement of the phase stability in atmospheres containing acid gases and an increase in the densification rate during sintering processes, which usually require very high temperatures for STF materials [36]. The permeation measurements reported in Figure 4 highlight that for *x* ≤ 0.4, the partial replacement of Sr by Ca in Sr_1-x_Ca_x_Ti_0.8_Fe_0.2_O_3−δ_ membranes does not lead to a substantial decrease in the oxygen separation capability. Therefore, Ca substitution can be considered a promising strategy to improve the stability and manufacturability of STF while keeping reasonable permeation properties.

### 3.2. Dual-Phase Materials

#### 3.2.1. Microstructure

**Figure 5:**
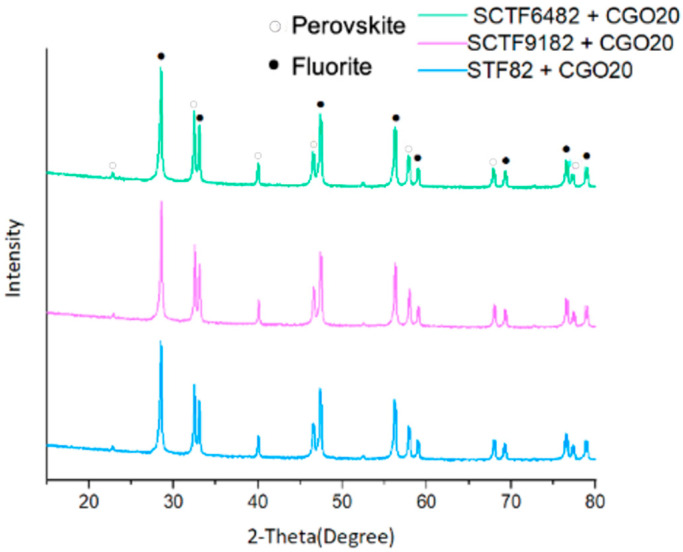
Diffractograms of sintered dual-phase membranes STF82 + CGO20, SCTF9182 + CGO20, and SCTF6482 + CGO20 at 1400 °C for 5 h.

The microstructure of dual-phase materials was investigated by SEM and XRD. XRD diffractograms (Figure 5) showed that all samples are composed only of the perovskite STF/SCFT and the fluorite CGO, confirming that no interaction occurred between the two materials during sintering.

**Figure 6:**
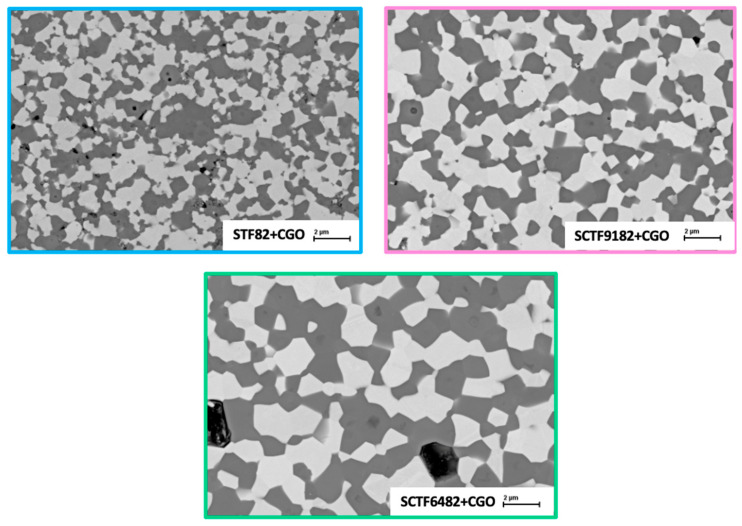
SEM-BSE images of dual-phase membranes STF82 + CGO20, SCTF9182 + CGO20, and SCF6482 + CGO20.

We investigated the phase distribution by SEM imaging. Figure 6 displays the cross-sectional back scattered electron (BSE) micrographs of the three membranes STF82 + CGO20, SCTF9182 + CGO20, and SCTF6482 + CGO20. All the materials consist of two phases. The lighter grains represent CGO20, while the darker ones are the perovskitic phase. By comparing the three images, it can be highlighted that the grain size systematically increases with Ca substitution in the order STF82 + CGO20 < SCTF9182 + CGO20 < SCTF6482 + CGO20.

**Figure 7:**
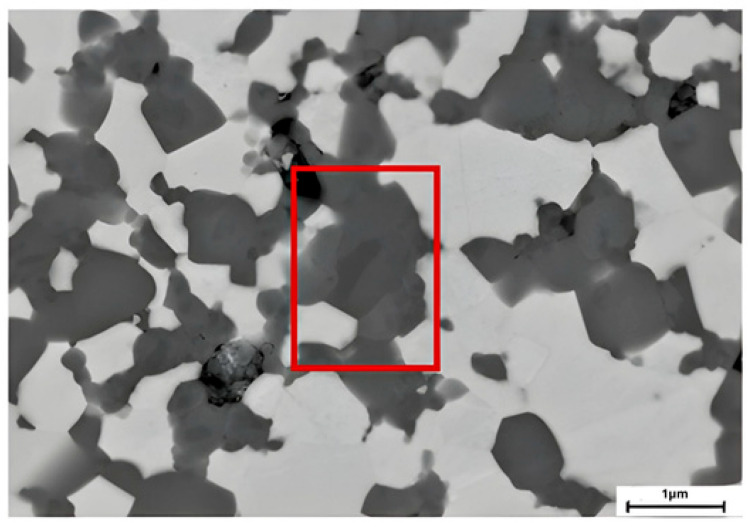
Detail of STF82 + CGO20 image where it is possible to see the presence of darker spots in the STF82 region.

SEM images at higher magnifications revealed the presence of darker spots on the perovskite grains in all the investigated compositions, exemplarily shown in Figure 7. This indicates inhomogeneities in the phase composition, but those could not be quantified either by SEM/EDS nor by XRD, so that no negative impact on performance or stability is expected.

#### 3.2.2. Oxygen Permeation Performance

**Figure 8:**
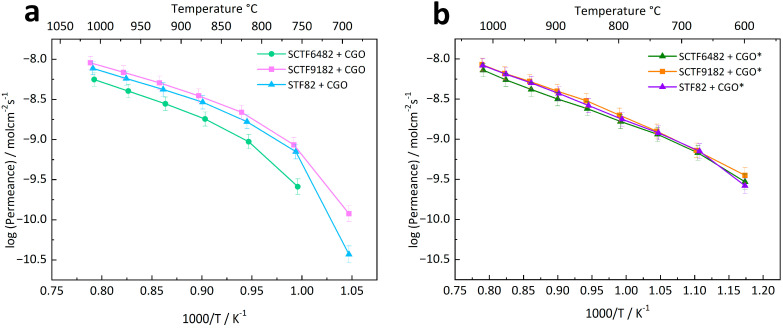
Permeance of (**a**) non-activated and (**b**) activated dual-phase membranes.

Figure 8a displays the permeance measured on the composite membranes STF82 + CGO, SCTF9182 + CGO, and SCTF6482 + CGO. In this case, the 40% Ca-substituted composite shows the lowest permeation efficiency. In contrast, the permeability of the 10% Ca-substituted composite is comparable to the non-substituted STF82 + CGO at temperatures higher than 700 °C. The activation energies of the three dual-phase membranes, calculated in the temperature range of 1000–800 °C, are reported in Table 3. The effect of the addition of CGO on oxygen transport properties can be highlighted by comparing the permeations measured on STF/SCTF + CGO samples (Figure 8a) with those observed for the corresponding single-phase STF/SCTF membranes (Figure 4). For STF82 + CGO and SCTF9182 + CGO, the oxygen fluxes are very similar to those observed in STF82 and SCTF9182, respectively. Moreover, the change in the activation energy is quite limited, respectively, + 4 and −10%, which can be reasonably ascribed to the experimental scattering of data. These results indicate that the addition of the ionic conducting fluorite to STF82 and SCTF6482 has a negligible effect on the permeation properties and, most likely, the oxygen permeation is controlled by the same mechanism both in single- and in dual-phase membranes.

On the other hand, the permeation of SCTF6482 + CGO is lower than that of bare SCTF6482, and the activation energy shows a significant increase of 28% (see Table 3), suggesting that the addition of CGO can impact the membrane’ oxygen separation efficiency. This behaviour could be related to the microstructure of the membrane itself. SEM images (Figure 6) show that SCTF6482 + CGO is characterized by larger grains than the other composites SFT82 + CGO and SCTF9182 + CGO. The different grain size could influence the oxygen dissociation/recombination reactions on the surface. All the composites are made of an ionic conductor, i.e., CGO, and a MIEC, i.e., STF. The surface region active for exchange reactions is therefore the whole perovskite area plus the TPB regions, where the two oxide phases and the air are in contact. Considering that the fluorite/perovksite ratio is the same for all samples, the total fraction of the membrane surface covered by the perovskite can be assumed to be equal for the three membranes. However, the coarser grain structure of SCTF6482 + CGO could result in a reduced extension of the TPB region and, consequently, in a reduced efficiency of the surface reactions. This would also explain the strong increase in the slope, i.e., activation energy, at lower temperatures in Figure 8a, which is typically assigned to surface exchange limitations. In addition, grain boundary effects at the fluorite–perovskite interfaces could affect the ionic transport in the bulk of the membranes and be related to the decrease in the permeation of SCTF6482 + CGO with respect to bare SCTF6482.

To investigate the effect of surface limitations in our dual-phase composites, a thin porous layer of La_0.6_Sr_0.4_Co_0.2_Fe_0.8_O_3−δ_ (LSCF), which is a good MIEC acting as a surface catalyst, was applied to both sides of the dual-phase membranes. This activation layer can extend the surface area available for the exchange reactions and improve the overall rate of oxygen dissociation and recombination.

Figure 8b displays, as an Arrhenius plot, the permeation measurements recorded on the activated membranes compared to the non-activated membranes in Figure 8a. It is possible to observe that the permeance of all three composites, once applied to the activation layer, is the same. The application of the LSCF catalyst also leads to a decrease in the activation energy in all samples (see Table 3), confirming that the permeation of the non-activated membranes is, to some extent, influenced by surface reactions. Moreover, the Arrhenius plots of activated samples are straight until low temperatures in contrast to those of non-activated ones, indicating that the application of the LSCF layer overcomes the surface exchange limitations even at a lower temperature. Interestingly, the activation energy calculated for each activated membrane is around 60 kJ/mol, which is in the range of CGO ionic conductivity [48]. Therefore, it is possible to suppose that the oxygen diffusion is limited by the bulk diffusion of oxygen ions through the membrane and, more specifically, through the fluorite phase CGO.

**Figure 9:**
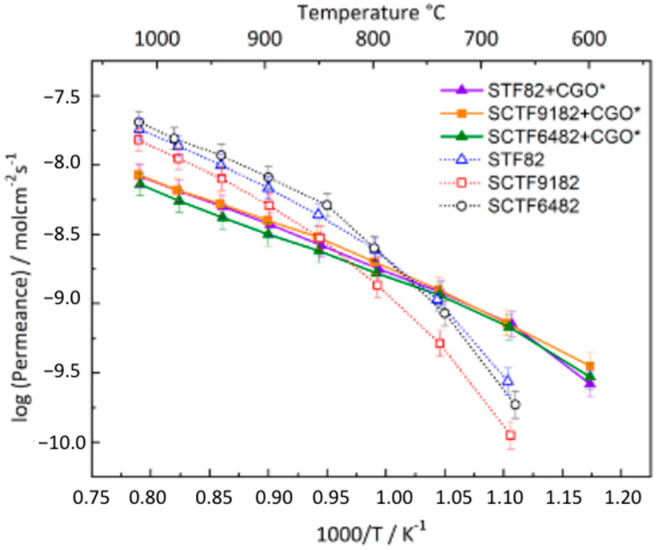
Permeance of dual-phase membrane with activation layer and single-phase membrane.

On the other hand, the permeance of the activated dual-phase membrane is still lower compared to the single-phase parent perovskitic membranes (Figure 9), so it is clear that the addition of CGO to STF/SCTF perovskites does not improve the overall ambipolar conductivity compared to the pure perovskites. Supposing that CGO is the predominant pathway for ionic conduction, it must be considered that, in the investigated dual-phase membranes, there is only a 60 wt% total of fluorite, which is equal to 51.7 v% volume percentage. Thus, it can be supposed that the amount of CGO is too low to confer any obvious benefit to the oxygen permeation of the perovskite single-phase membranes. However, an increase in such a percentage of fluorite ionic conductors might lead to better-performing dual-phase membranes, provided that the perovskite fraction is not lowered below the minimum threshold required to have percolation paths for electrons across the whole membrane thickness [49].

Once the most favourable fluorite/perovskite ratio is found, the permeation of the membranes can be further improved by reducing the thickness of the oxygen-selective layer. Asymmetric membranes composed of a thin (<30 µm) dense layer superimposed to a thick, porous support for mechanical stability are the most promising solution to achieve high oxygen fluxes [31]. In such structures, a stronger effect of the surface limitations is expected than for bulk membranes due to the reduced thickness of the oxygen-permeable layer. Therefore, a careful activation of the surface is mandatory to maximize the membrane performance.

## 4. Conclusions

In this study, we investigated the effect of Ca substitution levels in Sr_1-x_Ca_x_Ti_0.8_Fe_0.2_O_3−δ_ perovskites used as MIEC material in single-phase membranes and in combination with an ionic conductor in dual-phase membranes.

In single-phase membranes, the substitution of Ca on the A-site of Sr_1-x_Ca_x_Ti_0.8_Fe_0.2_O_3−δ_ has only a minor influence on the oxygen permeation performance. However, SCTF6482 with 40% substitution seems to be slightly more permeable than SCTF9182. At the same time, the introduction of Ca allows for a reduction in the sintering temperature of the membranes, facilitating membrane manufacturing. As such, the partial replacement of Sr with Ca can preserve the oxygen separation performance while conferring advantages in membrane processing and stability. Nevertheless, the intermediate range of substitution (0.1 < x_Ca_ < 0.4) should be studied in future work to obtain deeper insight into the effects of Ca substitution.

When the Sr_1-x_Ca_x_Ti_0.8_Fe_0.2_O_3−δ_ perovskites are combined with fluorite CGO in dual-phase membranes, the Ca fraction influences the microstructure, i.e., increasing Ca content leads to increasing grain size. Oxygen permeation is—at least partly—limited by surface-exchange reactions. This limitation can be overcome by the application of an activation layer made of La_0.6_Sr_0.4_Co_0.2_Fe_0.8_O_3−δ_ on the membrane surface. Activated STF82 + CGO, SCTF9182 + CGO and SCTF6482 + CGO have comparable permeation, which is limited by the ionic bulk transport in the fluorite phase. However, the performance of activated dual-phase samples still remains lower than single-phase perovskite membranes, possibly due to the low CGO amount. Therefore, mixing STF perovskite with ion-conducting CGO may be not a promising strategy to improve the oxygen permeation fluxes. To better clarify this point, in future studies, different fluorite/perovskite ratios must be investigated to find the most favourable one and compare it with single-phase STF membranes. After identifying the best dual-phase membrane composition, a further increase in the oxygen flux can be obtained by (i) the optimization of the membrane microstructure, e.g., developing asymmetric membranes, and (ii) the proper functionalization of the surface by coating an activation layer on the membrane.

## Figures and Tables

**Table 1:** Sintering condition for different composites.

Membranes	Temperature	Sintering Time
STF82	1450 °C	5 h
SCTF9182	1400 °C	5 h
SCTF6482	1350 °C	5 h
STF82 + CGO	1400 °C	5 h
SCTF9182 + CGO	1400 °C	5 h
SCTF6482 + CGO	1400 °C	5 h

**Table 2:** Activation energies of single-phase membranes STF82, SCTF9182, and SCTF6482 in the range of 800–1000 °C.

Membranes	Activation Energy kJ/mol
STF82	78
SCTF9182	87
SCTF6482	74

**Table 3:** Activation energies of the of dual-phase membranes and with an activation layer (which are marked with an asterisk) in the range of 800–1000 °C.

Membranes	Activation Energy kJ/mol
STF82 + CGO	81
SCTF9182 + CGO	78
SCTF6482 + CGO	95
STF82 + CGO *	61
SCTF9182 + CGO *	57
SCTF6482 + CGO *	61

## Data Availability

The data presented in this study are available on request from the corresponding author.

## Supplementary Materials

The following supporting information can be downloaded at: https://www.mdpi.com/article/10.3390/membranes15090258/s1, Figure S1: Rietveld refinement of X-ray diffraction (XRD) patterns recorded on sintered membranes composed by (a) STF82, (b) SCTF1982, and (c) SCTF6482.
