# Supplementary material for: Effects of Ca Substitution in Single-Phase Sr1-xCaxTi0.8Fe0.2O3-ẟ Oxygen Transport Membranes and in Dual-Phase Sr1-xCaxTi0.8Fe0.2O3-ẟ-Ce0.8Gd0.2O2 Membranes
Source: Membranes (Basel). 2025 Aug 29;15(9):258. doi: 10.3390/membranes15090258 (PMC12471747; doi:10.3390/membranes15090258)
Supplement: Supplementary file 1 [file membranes-15-00258-s001.zip › membranes-3726213-supplementary.pdf]

Supplementary Materials

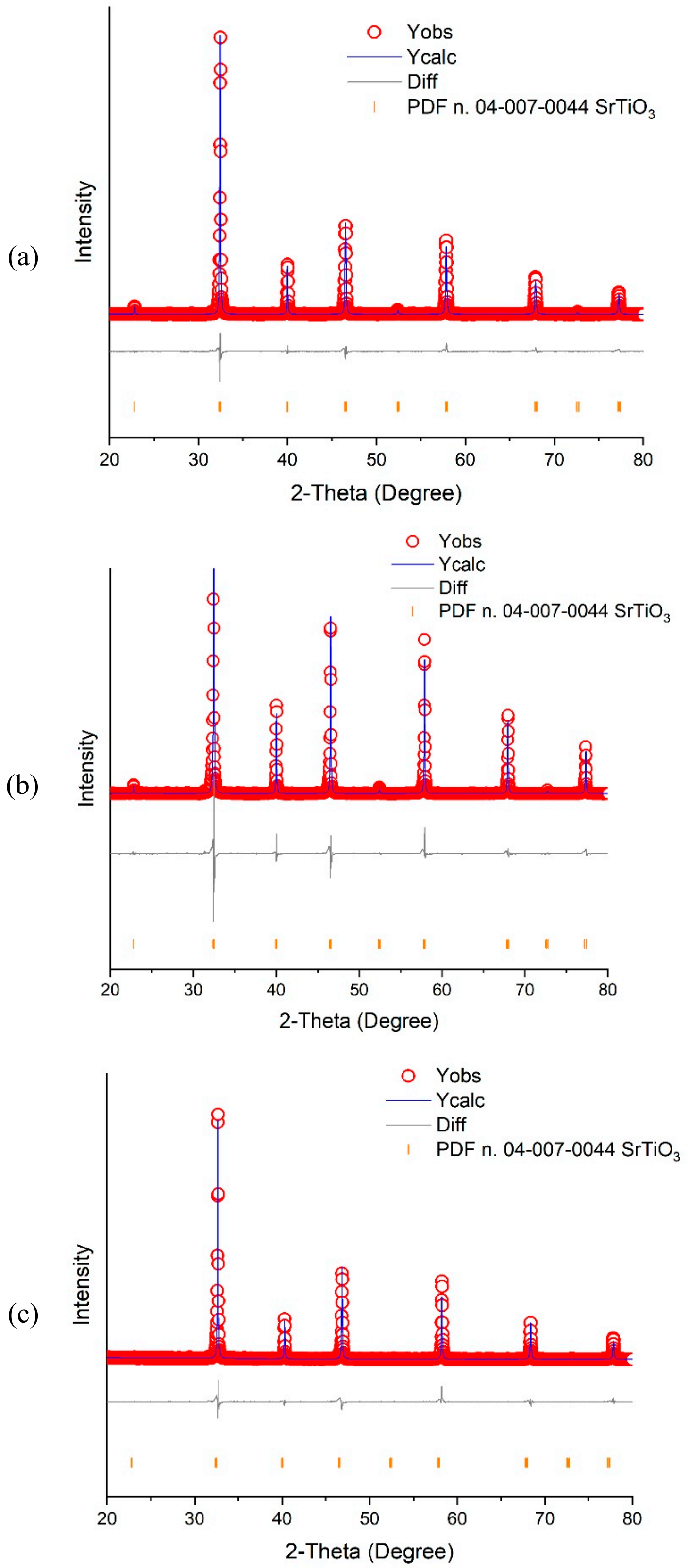

Figure S1 Rietveld refinement of X-ray diffraction (XRD) patterns recorded on sintered membranes composed by (a) STF82, (b) SCTF1982, and (c) SCTF6482.
